# Cultural transmission in a food preparation task: The role of interactivity, innovation and storytelling

**DOI:** 10.1371/journal.pone.0221278

**Published:** 2019-09-18

**Authors:** Lucas M. Bietti, Adrian Bangerter, Dominique Knutsen, Eric Mayor

**Affiliations:** 1 Institute of Work and Organizational Psychology, University of Neuchâtel, Neuchâtel, Switzerland; 2 Centre National de la Recherche Scientifique (CNRS), Télécom Paris, Institut Interdisciplinaire de l’innovation, UMR 9217, Paris, France; 3 Univ. Lille, CNRS, CHU Lille, UMR 9193—SCALab—Sciences Cognitives et Sciences Affectives, Lille, France; University of Edinburgh, UNITED KINGDOM

## Abstract

Interactive conversation drives the transmission of cultural information in small groups and large networks. In formal (e.g. schools) and informal (e.g. home) learning settings, interactivity does not only allow individuals and groups to faithfully transmit and learn new knowledge and skills, but also to boost cumulative cultural evolution. Here we investigate how interactivity affects performance, teaching, learning, innovation and chosen diffusion mode (e.g. instructional discourse vs. storytelling) of previously acquired information in a transmission chain experiment. In our experiment, participants (n = 288) working in 48 chains with three generations of pairs had to learn and complete a collaborative food preparation task (ravioli-making), and then transmit their experience to a new generation of participants in an interactive and non-interactive condition. Food preparation is a real-world task that it is taught and learned across cultures and transmitted over generations in families and groups. Pairs were defined as teachers or learners depending on their role in the transmission chain. The number of good exemplars of ravioli each pair produced was taken as measurement of performance. Contrary to our expectations, the results did not reveal that (1) performance increased over generations or that (2) interactivity in transmission sessions promoted increased performance. However, the results showed that (3) interactivity promoted the transmission of more information from teachers to learners; (4) increased quantity of information transmission from teachers led to higher performance in learners; (5) higher performance generations introduced more innovations in transmission sessions; (6) learners applied those transmitted innovations to their performance which made them persist over generations; (7) storytelling was specialized for the transmission of non-routine, unexpected information. Our findings offer new insights on how interactivity, innovation and storytelling affect the cultural transmission of complex collaborative tasks.

## Introduction

Human societies are shaped by cumulative cultural evolution, the cumulative improvement of cultural artefacts from one generation to the next, which is based on high-fidelity imitation and successive modifications of previously transmitted information and products [1]. In many cases, cumulative cultural evolution relies on the transmission of ‘recipes’ [2], which are composed of ingredients and instructions. Ingredients are the materials to design a new artifact whereas instructions are the behavioral rules that should be followed to make it. Like recipes, information and products are transmitted within and across social groups and communities. Hence, social learning [3, 4] and teaching [5] play a crucial role in the transmission and emergence of new products [6, 7, 8]. Social learning [4] is learning by observing or interacting with another individual or a product whereas teaching is “behavior evolved to facilitate learning in others” [5] enabling younger or less experienced group members to become better fitted to their community.

Interactive conversation drives the transmission of cultural information in small groups and large networks [9, 10, 11]. In formal (e.g. schools) and informal (e.g. home) learning settings, interactivity does not only allow individuals and groups to faithfully transmit and learn new knowledge and skills but also to boost cumulative cultural evolution. Research has shown that in the transmission of narrative texts [12] and route descriptions on a map [13] giving participants the opportunity to freely interact facilitates accurate information transmission. This is in line with research on human dialogue, which suggests that contributions to conversations enable dialogue partners to add information to their common ground–which is made of the knowledge that they share and are aware of sharing [14, 15]. This grounding process has been described as interactive, as it involves the active participation of all dialogue partners [16]. It enables people to accumulate mutual knowledge in both face-to-face and mediated communication [17, 18, 19].

But cumulative cultural evolution goes beyond the transmission of narrative texts. It encompasses the transmission of material culture (e.g. tools) and skills (e.g. cooking) which are often also supported by linguistic information. Experimental studies have used the method of serial reproduction [20, 21, 22] to simulate cumulative cultural evolution (building e.g. woven baskets, knots, paper airplanes, and stone tools) [3, 6, 23, 24]. This research has typically compared various modes of information transmission, including imitation (new generations observed what previous generations did), emulation (new generations observed cultural products and their performance) and teaching (new and old generations interacted about the completed task). In low complexity tasks—e.g. building a paper airplane or building a tower having as tools only spaghetti and modeling clay–[3, 23, 24], cumulative cultural evolution can occur in any of these conditions. That is, teaching is not necessary for cumulative culture to accrue. However, for more complex tasks—e.g. making stone tools–[6, 7], teaching promotes cumulative cultural evolution, compared to other forms of transmission. In this study we experimentally tested whether having the possibility for learners to interact with teachers in transmission sessions (1) promotes increased subsequent task performance of learners in transmission chains, (2) fosters the emergence and transmission of innovations, and whether (3) the transmission of innovations in teaching sessions was related to teachers’ previous performance.

Another aspect related to the quality of the transmission sessions, which we investigated further, was the role of storytelling in the transmission of information. The role of storytelling in the transmission of subsistence skills has rarely been tested in the laboratory before [25]. In its canonical form, storytelling is a collaborative conversational activity focused on the production of narrative discourse [26], whereby a narrator typically recounts a sequence of past events, including protagonists’ actions, and how they contribute to changing an initial situation [27]. Members of the audience participate in the activity by reacting to the stories being told or guiding them [28]. Experimental studies on the role of teaching in the transmission of simple [3] and complex [6] skills typically focussed on assessing the quality of the products produced by newer generations after transmission sessions. That is, they did not analyze the content of the dialogues between teachers and learners in teaching sessions. Storytelling is one way in which those contents are transmitted [29].

Teaching via storytelling constitutes a means for learners to vicariously expand their own experience via teachers’ experiences [20, 29], which in turn may enhance their ability to imagine or predict future events in relation to the task. Storytelling represents a key element in the creation and propagation of culture [25]. The universality of storytelling [30] suggests it may have an adaptive function, that is, it may have evolved because it confers some kind of fitness benefit to individuals or groups [25, 29, 31, 32]. However, while evidence from foraging [31, 32] and farmer [33] societies and results obtained in experiments [34] suggest that storytelling plays an important role in transmission of survival-related information (e.g. cultural information about ecology, religious belief and practices and cultural values and kinship), its exact role as a teaching method is unclear [25]. Other forms of communication (e.g., direct instruction) seem to be more frequent, and more efficient, in teaching than storytelling [32]. However, the specific adaptive value of storytelling lies in making sense of non-routine, uncertain or novel situations [25]. In this study we investigated whether (4) storytelling plays a specific role in the transmission of skills.

### Our experiment

**Fig 1:**
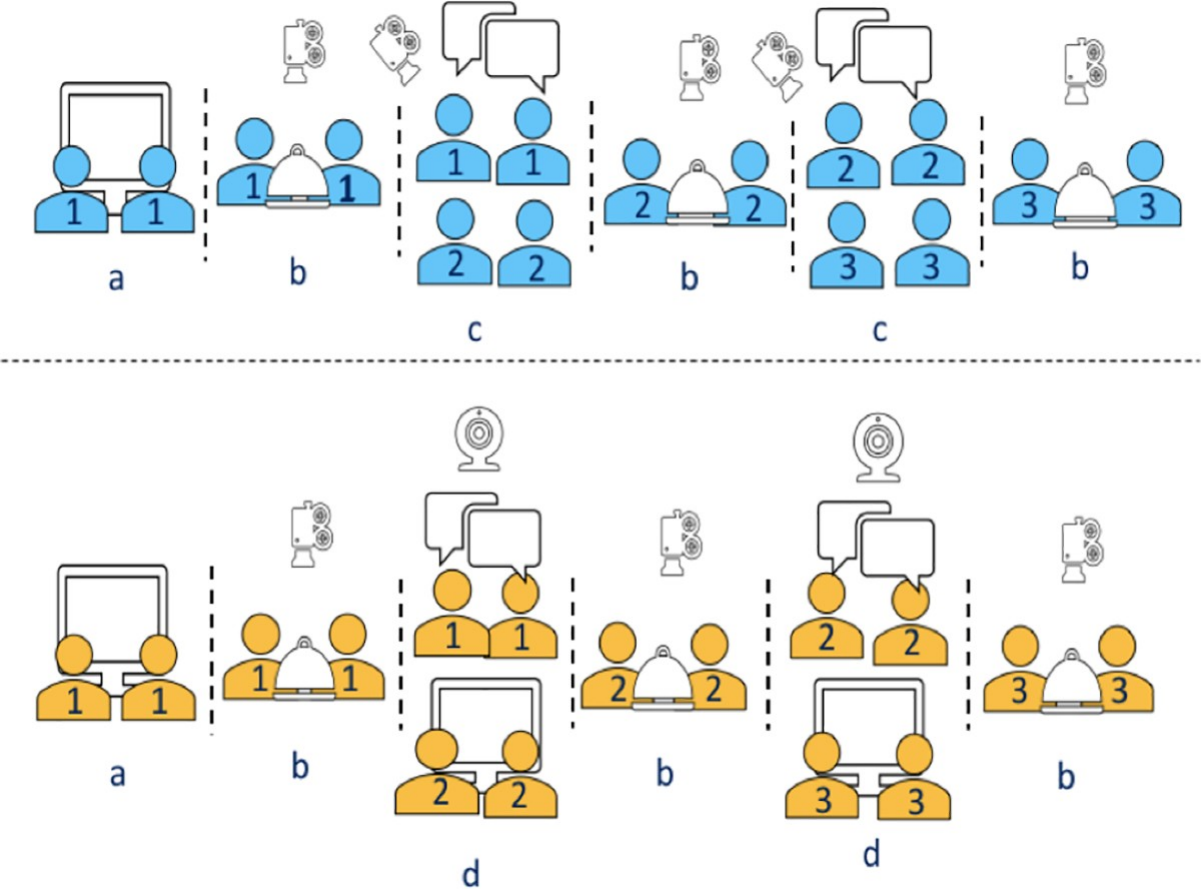
**Sequence of sessions in the experiment and generations involved in each session across interactive (blue) and non-interactive (orange) conditions**. **a**. Generation 1 (blue and orange) watched a 3 min 47 sec video tutorial on a computer screen that was recorded for the study. **b.** Generation 1, 2 and 3 (blue and orange) completed performance session 1, 2, and 3 respectively. **c.** Generation 1 (blue) transmitted their experiences to Generation 2 (blue) in a face-to-face conversation. Generation 2 (blue) followed the same procedure. **d**. Generation 1 (orange) had to video-record their instruction for Generation 2 (red) that then they watched on a computer screen. Generation 2 (orange) followed the same procedure. Performances in the interactive (blue) and non-interactive (orange) conditions lasted 10 minutes and afterwards an experimenter counted the number of ‘good quality’ ravioli they produced.

In this study we analyze how interactivity affects performance, teaching, learning, innovation and chosen diffusion mode (e.g. instructional discourse vs. storytelling) of previously acquired information in a transmission chain experiment. We adopted a transmission chain method [20, 21] to investigate how interactivity affects social learning, innovation and storytelling in a collaborative task in the laboratory. We conducted an experimental study (n = 288) on the cultural transmission of memories and skills collected from a collaborative food preparation task (ravioli-making) across transmission chains [35]. Under two conditions (interactive vs. non-interactive), pairs of participants transmitted memories and skills over three generations in 48 transmission chains. While in the interactive condition, transmission occurred in face-to-face conversations, in the non-interactive conditions, pairs video-recorded their instructions to the next generations (Fig 1). Our experiment thus investigated the effect of interactivity in the transmission of complex manual skills, for which teaching may often be considered necessary for its successful completion.

Food preparation is a social activity taught and learned across cultures and societies that currently attracts a lot of media attention. This is reflected in the increasing number of cookbooks that are sold annually, TV shows, online courses and tutorials available on the subject. Food preparation is a meaningful real-world task with well-documented evolutionary benefits. It enhances survival and nutritional fitness by reducing harmful bacteria and increasing the digestibility of food [36, 37]. It can boost creativity [38], and have a positive impact on people’s self-esteem [39]. When food preparation occurs collaboratively it strengthens social bonds by reinforcing family relationships, initiating and underpinning friendship [40], Longitudinal studies have shown that individuals who were able to develop sufficient cooking and food preparation skills by emerging adulthood tend to be healthier and have better nutrition [41].

Our methodological choice for the investigation of the effects of interactivity on the transmission of memories and skills over three generations was in line with previous studies focused on the influence of social interaction in cultural transmission. For instance, Tan and Fay [12] used 16 four-person transmission chains to examine the effects of interactivity on the transmission of verbal information. In their study they had one participant per generation and interactive transmissions were limited to two participants. In our study, 48 transmission chains contained six participants each, having two participants per generation and four participants in interactive transmission sessions. The complexity of the task, its duration (10 minutes) and the fact that transmission sessions did not have time constrains were additional factors that made us restrict the number of generations to three in order to ensure overall feasibility.

**Fig 2:**
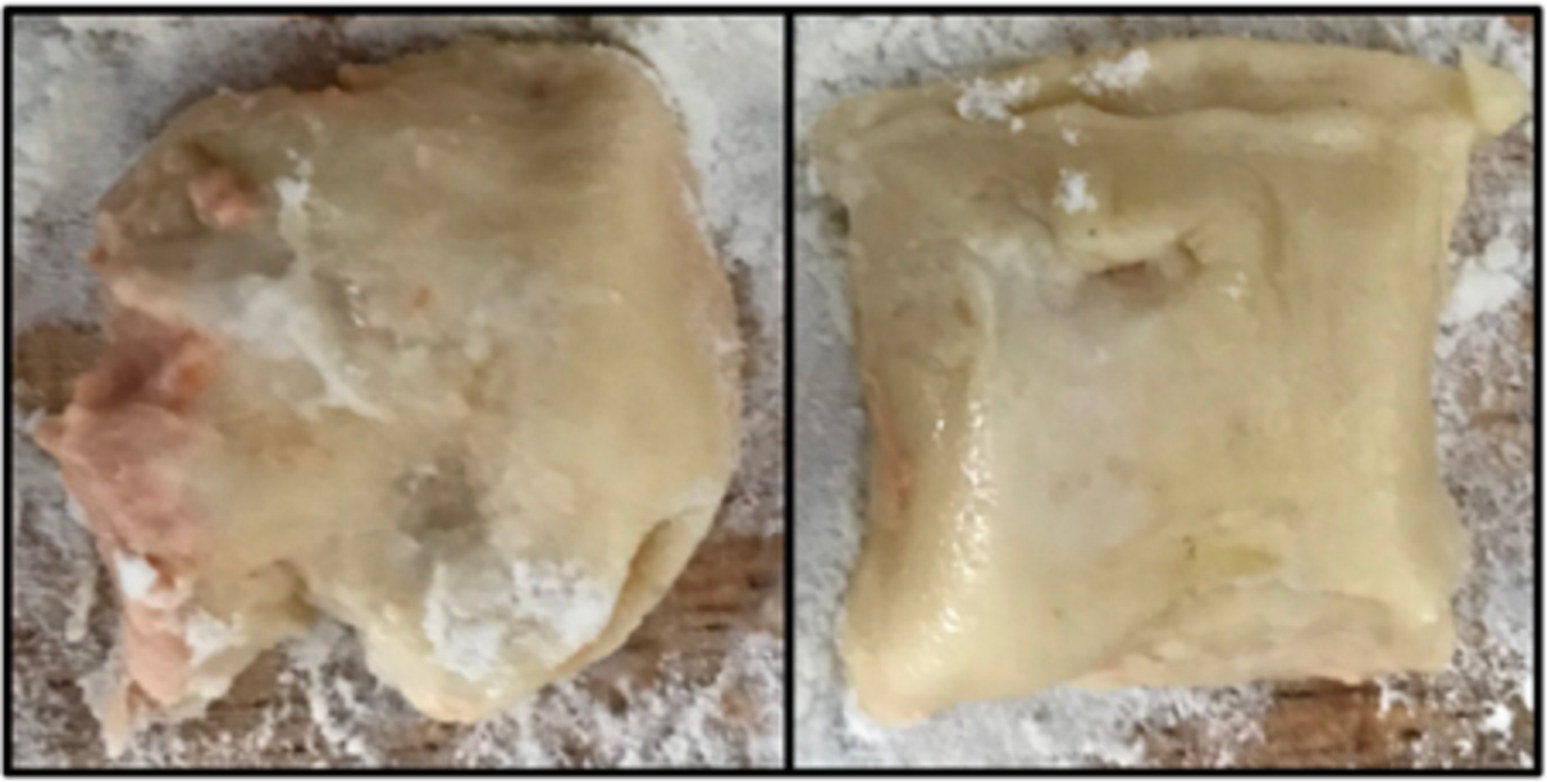
**Examples of low quality (left) vs. high quality (right) ravioli**. Only the number of high-quality ravioli was counted to measure performance in the collaborative task. Tomato concentrate paste was used to facilitate the detection of leaks in the dough.

In the experiment we used an instructional video as starting point which gave Generation 1 information about the steps they should follow to complete the collaborative food preparation task (Fig 1). Task performance was measured by counting the total number of ‘good quality’ ravioli produced by each pair (Fig 2). However, anecdotal evidence collected in several pilot studies using the same task and population (individuals with limited cooking experience) showed that some subjects could not complete basic steps due to lack of essential culinary knowledge (e.g., use of flour to keep dough from sticking). We thus decided to use an instructional video as starting point in order to ensure that most people had a minimum of basic knowledge.

The first set of hypotheses (H1-H2; see section below: Effects of generation and interactivity on task performance) we tested were specifically related to how transmissions over generations and interactivity in transmission sessions affect task performance. The second set of hypotheses (H3-H4; see section below: Effect of information quantity on task performance) examined whether the quantity of information conveyed in transmission sessions affects task performance and how this might have been influenced by interactivity. In order to test the third (H5-H8; see section below: The emergence and transmission of innovation) and fourth (H9-H11; see section below: The role of storytelling as teaching method) sets of hypotheses we examined the actual characteristics of the information transmitted over generations. To do so, we conducted additional analyses on specific features of the verbal protocols.

### Effects of generation and interactivity on task performance

In line with studies using manual tasks [3, 23, 24], we expected that performance will improve over generations due to the accumulation of learned improvements. Performance was measured in number of ‘good quality ravioli’ (Methods). We further expected interactive transmissions to allow learners to ask questions and request clarifications [9], and thus, to stimulate teachers to talk more and provide additional information. This in turn, may lead to a better transmission of skills. As a result, we also expected interactivity to lead to better performance than non-interactivity in transmission sessions. These were the hypotheses we tested: (H1) performance improves over generations; and (H2) performance improves more due to interactivity.

### Effect of information quantity on task performance

The second set of hypotheses (H3-H4) investigated the effects of information quantity on task performance in interactive and non-interactive transmission. Learners’ possibility to freely interact with teachers in the interactive condition would be associated with the transmission of more information from teachers. Teachers would not only have to instruct learners to successfully complete the task but also to respond to questions and give clarifications in order to ensure mutual comprehension. One of the main features of collaborative dialogue is that dialogue partners must make sure that they have understood each other before they can move on to the next contribution [15, 16]. This may cause teachers to increase the quantity of information transmitted. Thus, we expected longer transmissions to lead to subsequent higher performance in learners when compared to performances following shorter transmissions. As result we tested whether (H3) teachers transmit more information during interactive transmissions; and if (H4) performance is predicted by the amount of information in the preceding transmission session.

### The emergence and transmission of innovation

**Fig 3:**
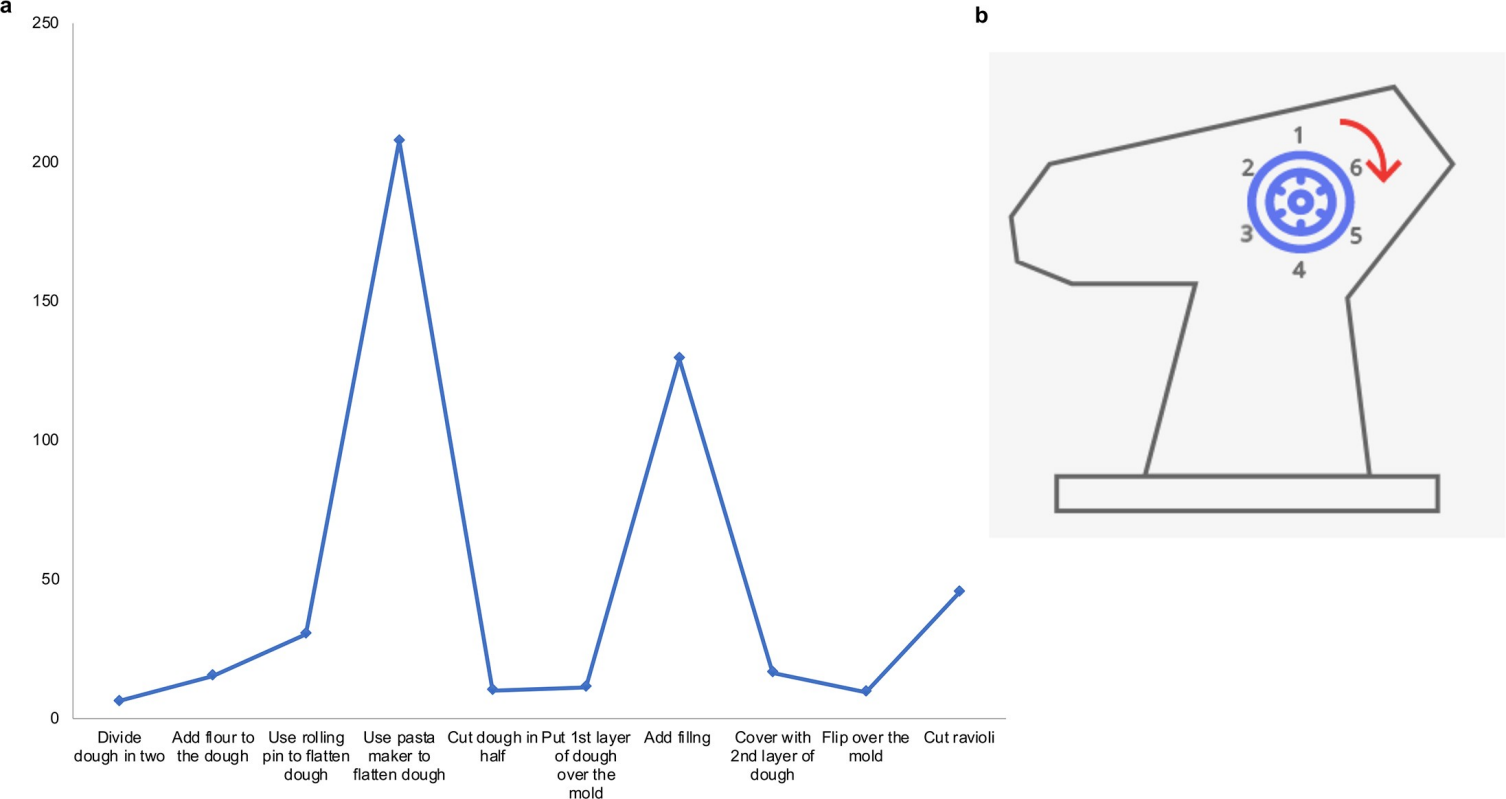
a. Mean duration of 10 phases of the collaborative task. b. Drawing of the knob participants had to turn to switch the levels of the machine to make the dough gradually thinner.

The third set of hypotheses (H5-H8) investigated was related to the effects of cultural transmission on the emergence and diffusion of innovation. These hypotheses were tested in order to provide possible explanations for any improvements in task performance over generations. In cultural evolution research [1] innovation refers to the proposal of a new variant, which may occur through a novel invention, recombination, refinement and exaptation [42, 43]. Due to the constraints given by the experiment, here we concentrated on the emergence of a new variant through refinement only, that is, the modification or improvement of existing alternatives. Refinement generally occurs in a goal-oriented fashion. In particular, we investigated how teachers transmitted information about the use of the pasta maker to flatten the dough (Methods). This was the longest phase of the task for pairs in both conditions (Fig 3A). Hence, saving time in this phase was an important incentive that pairs had for the successful completion of the remaining phases of the task (e.g. adding filling) within the allocated 10 minutes. In addition, this was also the least intuitive phase of the task to complete based on informal debrief with participants following the experiment. The information that Generation 1 received from the video tutorial (Fig 1) indicated that pairs had to pass the dough through the pasta maker six times (up to level six, Fig 3B). An instance of innovation could appear when, in transmission sessions, teachers mentioned the possibility of using fewer levels of the pasta maker either by not reaching level 6 (e.g. 1–5) or skipping levels (e.g. 1, 3, 5 and 6) in order to save time. The nature of the task was based on copying what the previous generation recalled having done in each step of the collaborative activity. Thus, the task did not allow generations to innovate beyond the use of the pasta maker to flatten the dough. We expected that the innovation would be transmitted in teaching sessions, and lead to increased performance in learners. This may occur because generations would have more available time to spend on other important phases of the task (e.g. cutting ravioli).

We also analyzed whether there was an association between the inclusion of innovations in transmission sessions and previous performance, that is, if there was a correlation between the number of good quality ravioli produced and the transmission of innovations. It could have been the case that teachers had enough time to refine a new variant during the collaborative task, apply it to their own performance and then transmit it to learners. The new variant could have also been refined after the completion of the task when realizing that the rule they followed was not effective. We conducted an additional analysis on whether learners followed these suggested innovations while completing the collaborative task. In particular, the hypotheses we tested were: (H5) the transmission of innovation increases over generations; (H6) previous performance of teachers predicts the inclusion of innovation in their transmissions; (H7) learners follow the innovation transmitted by teachers; and (H8) innovation in teaching sessions predicts increased performance in learners.

### The role of storytelling as teaching method

The fourth set of hypotheses (H9-H11) investigated was also related to the quality of the transmission sessions. We analyzed the role of storytelling in the transmission of information. Recent research [25] has argued that the adaptive value of storytelling lies in making sense of non-routine, uncertain or novel situations, which consist in events either turning out better than expected, or not living up to expectations. In order to empirically examine whether storytelling was specialized for transmitting non-routine or unexpected information about the task compared to instructional discourse, we tested if (H9) teachers preferentially use storytelling to transmit non-routine or unexpected information. In addition, we tested whether (H10) teachers’ storytelling increases over generations; and (H11) whether teachers’ storytelling is facilitated by interactivity. The relationship between the presence of storytelling and increased performance was included in the analysis of innovation (Methods: measures).

## Methods

### Participants

Participants (n = 288; 132 men) were recruited from the student population of the University of Neuchâtel (Age M = 23.2; SD = 4.07). They were fluent speakers of French and reported having limited previous cooking experience. They had previous practice of simple skills like combining and heating ingredients but had not mastered more complex skills (e.g., preparing a pie from scratch). Participants received 25 CHF compensation each for half an hour of their time along with an incentive of 0.25 CHF per pair for each produced ravioli of good quality (Fig 1). There were 48 chains (24 in the interactive condition and 24 in the non-interactive condition). Pairs of participants were randomly assigned to different conditions (interactive vs. non-interactive) and chain within condition (1–24) but the assignment to generation was non-random as we ran the next available generation (1–3) in the chain (Fig 1). Three chains from the interactive condition had to be removed for technical reasons. Thus, the data that were analyzed included 45 chains (21 in the interactive condition and 24 in the non-interactive condition).

### Task

The task consisted of two kinds of sessions, performance sessions and transmission sessions (Fig 1). In performance sessions, participants from each generation prepared ravioli together in pairs. Their goal was to produce as many good quality ravioli as possible in 10 minutes. Each pair had at their disposal a ball of 150 grams of dough; 200 grams of filling made of ricotta cheese and concentrated tomato paste (for easy detection of leaks and imperfections when evaluating ravioli quality); a 24-hole ravioli mold with zigzag sealing for easy release; a pasta maker; a rolling pin; a cutting board; 2 pizza cutters; 2 knives; 4 teaspoons; 2 kitchen cloths and kitchen paper; 250 grams of flour; and a stopwatch. Immediately after the time was up, the ravioli were assessed by the experimenter (Methods: measures). Transmission sessions occurred immediately after each performance session. Pairs who had just completed the task explained to next-generation pairs how to prepare the ravioli. Explanations were either face-to-face (interactive condition) or video-recorded (non-interactive condition). Video recordings were then watched by incoming generations. These sessions were unstructured and did not have time constraints (they typically lasted between 2 and 7 minutes).

### Design

The experimental design we used was the vertical transmission chain method [3, 6, 22, 23, 24], manipulating the participants’ possibilities to freely interact during transmission sessions and adapting it for collaborative tasks. Chains of three generations of participants made ravioli in pairs and transmitted their experience to a pair in the next generation. This occurred under two conditions (interactive condition vs. non-interactive condition). In the interactive condition, transmissions occurred in face-to-face conversations, whereas in the non-interactive condition they were video-recorded as instructions to the next generation (Fig 1).

### Procedure

Participants signed consent forms upon their arrival. Generation 1 watched a 3 min 47 sec video tutorial that was recorded for the study (Fig 1). It provided information about the steps to be followed to prepare ravioli in pairs. They then completed Performance session 1, followed by Transmission session 1 (together with Generation 2 in the interactive condition or through video recording in the non-interactive condition). Then, Generation 2 completed Performance session 2. During this time, Generation 1 pairs were paid, debriefed and allowed to leave. After having completed Performance session 2, Generation 2 participated in Transmission session 2 (together with Generation 3 in the interactive condition or through video recording in the non-interactive condition). Then, Generation 3 completed Performance session 3. During this time, Generation 2 were paid, debriefed and allowed to leave. After Performance session 3, Generation 3 were paid, debriefed and allowed to leave.

### Measures

Different measures were used to test our hypotheses. In the description below, we explain each of our measures in relation to their role as dependent variable (DV).

#### Hypotheses 1–2

For H1 (Performance improves over generations) and H2 (Performance improves more due to interactivity) performance was measured as the quantity of ‘good’ ravioli (DV) each pair produced. The criteria that experimenters applied to consider ravioli as “up to a satisfactory standard” were that they should contain filling and they were perfectly sealed. We chose the filling to be red so any leaks in the ravioli would be immediately visible to experimenters (Fig 2). Experimenters used their fingers to gently press each of them in order to confirm that ravioli did not contain any leaks when this was not evident at first sight. Ravioli with leaks were considered as ‘low quality ravioli’ and discarded. The rationale for having adopted these criteria was twofold: (i) ravioli should be perfectly sealed so that they kept their shape and filling during boiling; and (ii) adding no filling would have made the ravioli easier to manipulate and would have enabled pairs to save time during the “adding filling” stage of the task (Fig 3A). In practice, it was easy to make the distinction between good and bad-quality ravioli.

#### Hypotheses 3–4

For testing H3 (Teachers transmit more information during interactive transmissions) and H4 (Performance is predicted by the amount of information in the preceding transmission session), information quantity (DV) was measured by the number of words uttered by teachers in transmission sessions. In order to do so, all sessions were videotaped and transcribed.

#### Hypotheses 5–8

To test H5 (The transmission of innovation increased over generations) we measured innovation (DV) in transmission sessions. To do so, we watched the videos of transmission sessions and examined the transcriptions. The video tutorial that Generation 1 watched before Performance 1 (Fig 1) instructed them to carefully pass the dough through the pasta maker, which had two rollers that could be adjusted using the knob on the side. This allowed making the dough gradually thinner. The video tutorial instructed Generation 1 to pass the dough six times (1, 2, 3, 4, 5, and 6) so as to obtain the right thickness of the dough (Fig 3B). It showed that each time before passing the dough through the pasta maker, generations had to turn the knob in order switch levels (6 levels).

We defined innovation as the teachers’ transmission of information about skipping levels of the pasta maker in order to save time. We followed two steps to measure innovation: i) we measured teachers’ inclusion of memory utterances (MUs) in transmission sessions to report what they actually did with the pasta maker to flatten the dough in previous performance sessions (Measure 1.1, Table 1); and) we coded all instances when in transmission sessions teachers included explicit advice on the specific use of the pasta maker for learners to achieve the most suitable pasta (“we recommend you using levels 2, 4 and 6 only”) (Measure 1.2, Table 1). We defined MUs as past-tense utterances generated by the teachers in the transmission sessions that were thematically related to the task [44]. Any deviation from rule as introduced in the video instruction (use levels 1, 2, 3, 4, 5 and 6) in MUs (e.g., “we passed the dough through levels 1, 2, 3 and 4”), advice (e.g., “we suggest you do only levels 2, 4, 5 and 6”) or both observed in teaching sessions was considered an instance of innovation. We assigned a score to each case of innovation. The innovation score was obtained by subtracting the number of levels that teachers recalled/recommended in transmission sessions (e.g., 1, 2, 3 4, 5 or 6 levels) from the number of levels introduced in the video tutorial (six levels). For example, if teachers recalled having used or recommended using the six levels of the pasta maker to flatten dough (“we did levels 1, 2, 3, 4, 5, and 6” / “we recommend you using levels 1, 2, 3, 4, 5 and 6”) the innovation score was zero because the was no deviation from the rule introduced in the video instruction (six levels in the video tutorial minus six levels in the MUs or advice equals zero as innovation score). Innovation score increased if teachers remembered having used or suggested to use less levels of the pasta maker. For instance, innovation score was one instead of zero if teachers recalled having used or recommended using five (e.g., 1, 2, 4, 5, 6) instead of six levels (six levels in the video tutorial minus five levels in MUs or advice equals one as innovation score). Innovation score increased as the recalled or recommended levels that teachers transmitted to learners decreased.

**Table 1:** Description of linguistic variables. Illustrative items are in boldface.

Measures	Example
**1. Innovation** 1.1 MUs in rule transmission only -Transmission of the rule from teachers to learners in the form of memory utterances. 1.2 Advice in rule transmission only -Transmission of the rule from teachers to learners in the form of advice.	[00:35.1] C1G1P2: And then suddenly that the uh it makes it really flat and you'll have to increase the volume so it goes from 1 to 6 so you have to increase gradually **but we've seen that if we went from 1 to 3 that's enough**. [00:38.7] C1G14P2: [….] You have to put the dough in a machine, to flatten it a bit. And uh you have to go until 6. **So uh, you have to go from 1 to 6, it's like a kind of level of thickness, we can say. And we recommend you not going from 1 to 6, but do it like 1, 3** [00:59.1] C1G14P1: **1, 3, 5**. [00:59.9] C1G14P2: **3, 5 and 6**.
**2. MUs in entire transmission sessions (Storytelling)** - Use of storytelling in the form of MUs to transmit information about all phases of the task.	[01:21.7] C2G23P3 […] In the end **we changed the roles when we passed the dough through the machine**. I let the machine flatten the dough with the rollers. **You did, you took the dough when it was quite long in fact**. **We had cut the dough, in fact not in the middle but longitudinally, so we had two bands.**
**3**. **Instructional discourse in entire transmission sessions (Non-MU)** - Use of instructional discourse to transmit information in entire transmission sessions about all phases of the task.	[01:01.5] C2G2P2: **There is a person who turns and the other who recovers the dough underneath**, and in fact **this allows to flatten the dough**, so it's useless to keep doing it too much with the rolling pin. The machine will also do this job […].
**4. Transmission of non-routine, unexpected information in MUs (entire transmission sessions)** - Use of MUs to transmit non-routine, unexpected information about all phases of the task in MUs (entire transmission sessions).	[01:24.5] C1G112P2: […] And very important what we were told to do **but we forgot to have to sprinkle flour to the ravioli mold to make it easier to unmold. We didn't, so we didn't manage to unmold the ravioli**.
**5**.**Transmission of non-routine, unexpected information In instructional discourse (Non-MU) (entire transmission sessions)** - Use of Non-MU to transmit non-routine, unexpected information about all phases of the task.	[01:34.4] C1G15P2: Suddenly you put the dough on it, you put the filling, **but not put too much filling because otherwise after it'll overflow.** [01:37.1] C1G15P1: **Well, they aren't counted**. She'll check if they sealed, or not, if it comes out. [01:44.7] C1G15P2: **It's not counted**. [01:45.3] C1G15P3: OK.

When teachers transmitted innovation both via MUs and advice, we considered the most innovative variant only, that is the variant that included fewer levels and resulted in higher innovation score. For example, if in the transmission session (G1-G2) Generation 1 recalled having put the dough through levels 1, 2, 5 and 6 (six levels in the video tutorial minus four levels in the MUs equals two as innovation score) and then recommended Generation 2 only using levels 1, 3 and 6 to do it faster (six levels in the video tutorial minus three levels in advice equals three as innovation score), we only counted advice in the coding.

To test H6 (Previous performance of teachers predicted the inclusion of innovation in their transmissions) we used previous performance, as we looked at how previous performance of teachers predicted the inclusion of innovation in their transmissions. H7 (Learners followed the innovation transmitted by teachers) was tested by using follow innovation as DV. Follow innovation was considered as learners’ tendency to follow teachers’ transmitted innovations (as explained for H5 above). In transmission sessions teachers always referred to the rule as introduced in the video tutorial (use 1, 2, 3, 4, 5 and 6 levels of the pasta maker to flatten the dough) and sometimes referred to their own experience in the form of MUs and give recommendations in the form of advice. We measured follow innovation by coding the number of times learners put the dough through the pasta maker to flatten the dough. When learners skipped more levels of the pasta maker than those transmitted by teachers this was also coded as a follow innovation, Thus, we computed whether learners followed (or not) the innovations proposed by teachers in transmission sessions. And finally, to test H8 (Innovation in teaching sessions predicted increased performance in learners) we calculated whether performance (DV) in learners increased due to received innovation.

#### Hypotheses 9–11

In order to test H9 (Teachers preferentially use storytelling to transmit non-routine or unexpected information) we coded all transmission sessions for whether or not teachers included MUs (see definition above) or instructional discourse (Non-MU) related to the task. In this context storytelling was characterized as the transmission of MUs in teaching sessions. Here the coding of MUs (Measure 2, Table 1) refers to the entire transmission sessions, not only to the part where teachers conveyed their own experience about how many levels of the pasta maker they used to flatten the dough (Innovation: Measure 1.1, Table 1). We defined Non-MU (Measure 3, Table 1) as talk referring to the transmission of procedures and facts, which aided the successful completion of the task (e.g., “one must add flour to the mold”). The transmission of information from teachers to learners occurred via MUs and Non-MU. However, Non-MU was the most chosen form to transmit information over generations in the interactive and non-interactive conditions. To measure MUs and Non-MU we followed a procedure similar to one that was used to measure information quantity (word count, see H3 and H4 above). Then, we coded MUs and Non-MU as either routine or non-routine. While routine MUs and Non-MU described mundane and expected events (e.g., “we flattened the dough with the rolling pin”), non-routine MUs (Measure 4, Table 1) and non-routine Non-MU (Measure 5, Table 1) depicted events not in line with expectations—either turning out badly or being surprisingly easy to do (e.g., “when we flipped the mold over the ravioli didn’t fall out”). We calculated MUs and Non-MU density by dividing the teachers’ raw MU and Non-MU word count by the total amount of words they spoke in transmissions. To test H10 (Teachers’ storytelling increases over generations) and H11 (Teachers’ storytelling is facilitated by interactivity) we used MUs (DV) as the continuous variable to measure whether storytelling increased over generations (H10) and whether it was facilitated by interactivity in transmission sessions (H11). Five observations were discarded from further analysis because teachers opted for the transmission instruction in the narrative form exclusively.

Twenty-five percent of transmission sessions were double-coded. Inter-rater agreement for all variables (S1B Table) was excellent (kappas > = .97). Disagreements across coders were resolved through discussion. Extracts from the corpus and the matching coding procedure are provided in Table 1. Examples were taken from transmission sessions.

## Results

**Fig 4:**
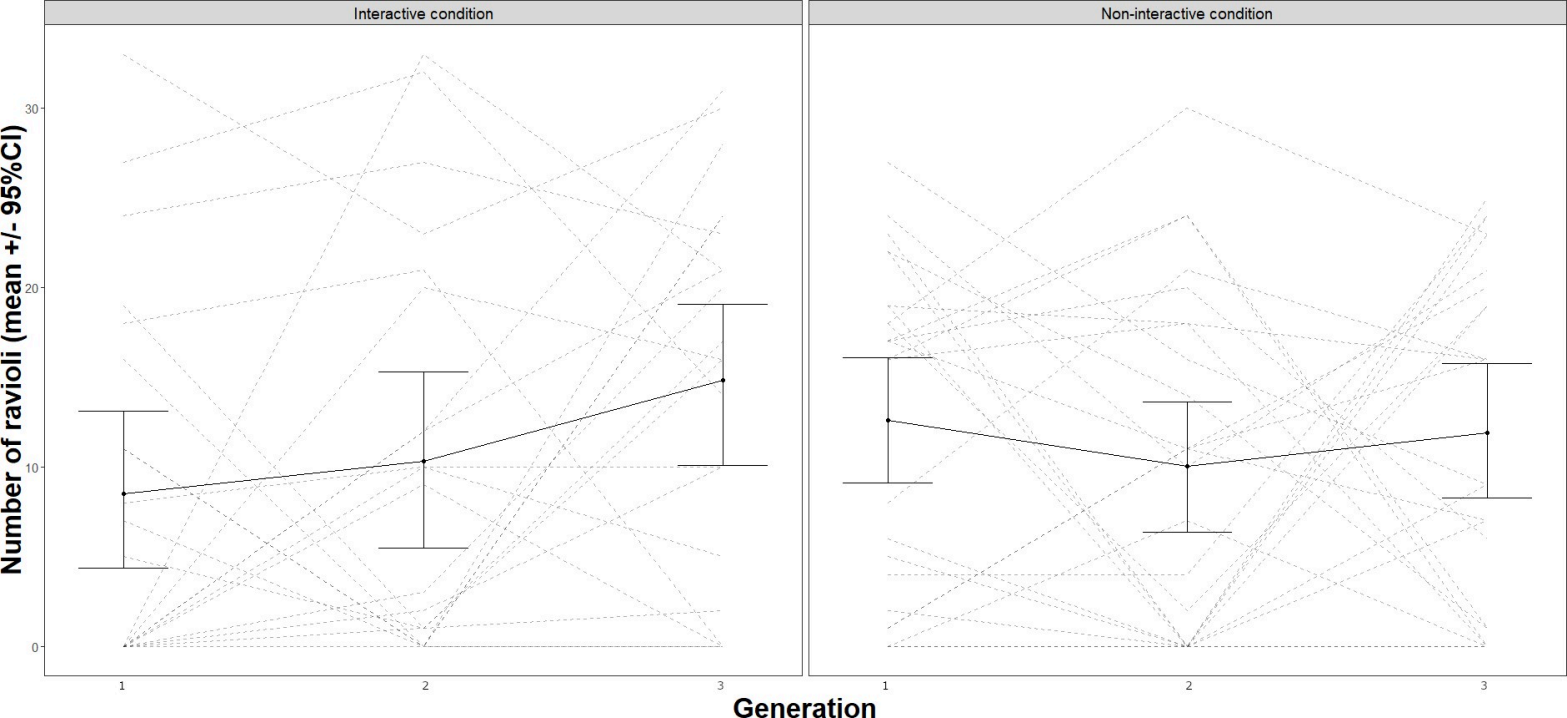
Performance in number of good quality ravioli produced by each generation (G1, G2, and G3) in interactive and non-interactive conditions. Error bars represent standard error to the mean.

**Fig 5:**
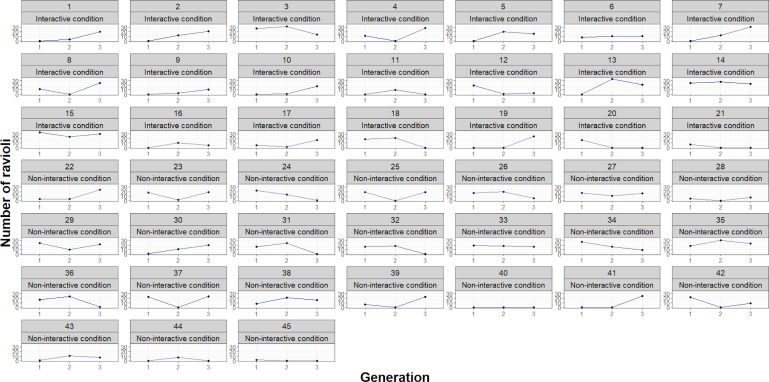
Each chain’s (n = 45) progression in terms of performance over generations (G1, G2, and G3). Chains 1–21 are in the interactive condition and chains 22–45 are in the non-interactive condition.

**Fig 6:**
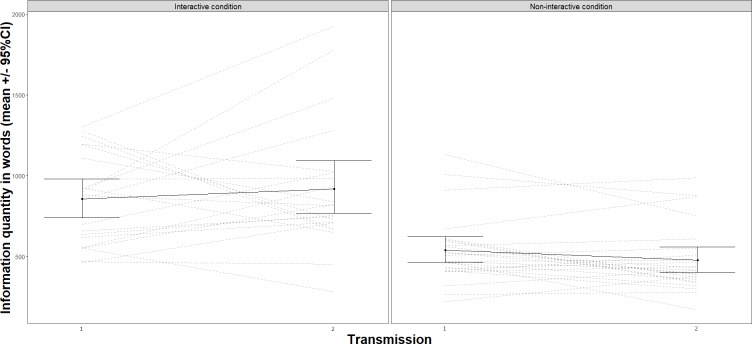
Number of words produced by teachers in transmission sessions G1-G2 and G2-G3 in interactive and non-interactive conditions. Error bars represent standard error to the mean.

We tested H1 (Performance improves over generations) and H2 (Performance improves more due to interactivity) by analyzing the effect of generation and interactivity on performance. The model used to test H1 and H2 (Analysis 1, S1B Table) included generation and interactivity as categorical variables as fixed effects and performance as the outcome. We found that performance did not improve over generations *F*(2,86) = 1.58, *p* = 0.212, or due to interactivity, *F*(1,43) = 0.02, *p* = 0.89 (Fig 4). We were thus unable to confirm our original predictions for H1 and H2 (See Fig 5 for performance in all transmission chains). However, we observed a significant effect of interactivity for H3 (Teachers transmit more information during interactive transmissions), *F*(1, 43) = 27.50, *p* < .001. The model used to test H3—effect of generation and condition on information quantity—included the same categorical variables as fixed effects and information quantity as the outcome (Analysis 2, S1B Table). This shows that teachers spoke more with co-present learners than to a video camera (Fig 6). Although we did not find an effect of interactivity on performance, this result suggests that teachers behaved differently depending on the presence or absence of listeners. Teachers transmitted more information in the interactive condition.

The model used to test the effect of generation, condition, and information quantity on next-generation performance contained categorical variables as well as two continuous variables as fixed effects (information quantity and innovation) and performance as the outcome (the same model was used to test H8, see above; Analysis 3, S1B Table). For H4 (Performance is predicted by the amount of information in the preceding transmission session), we found a significant effect of information quantity on next-generation performance *F*(1, 72) = 5.17, *p* = .026. That is, learners’ next-generation performance increased with information quantity transmitted by teachers, *b* = 3.01, *SE* = 1.32.

**Fig 7:**
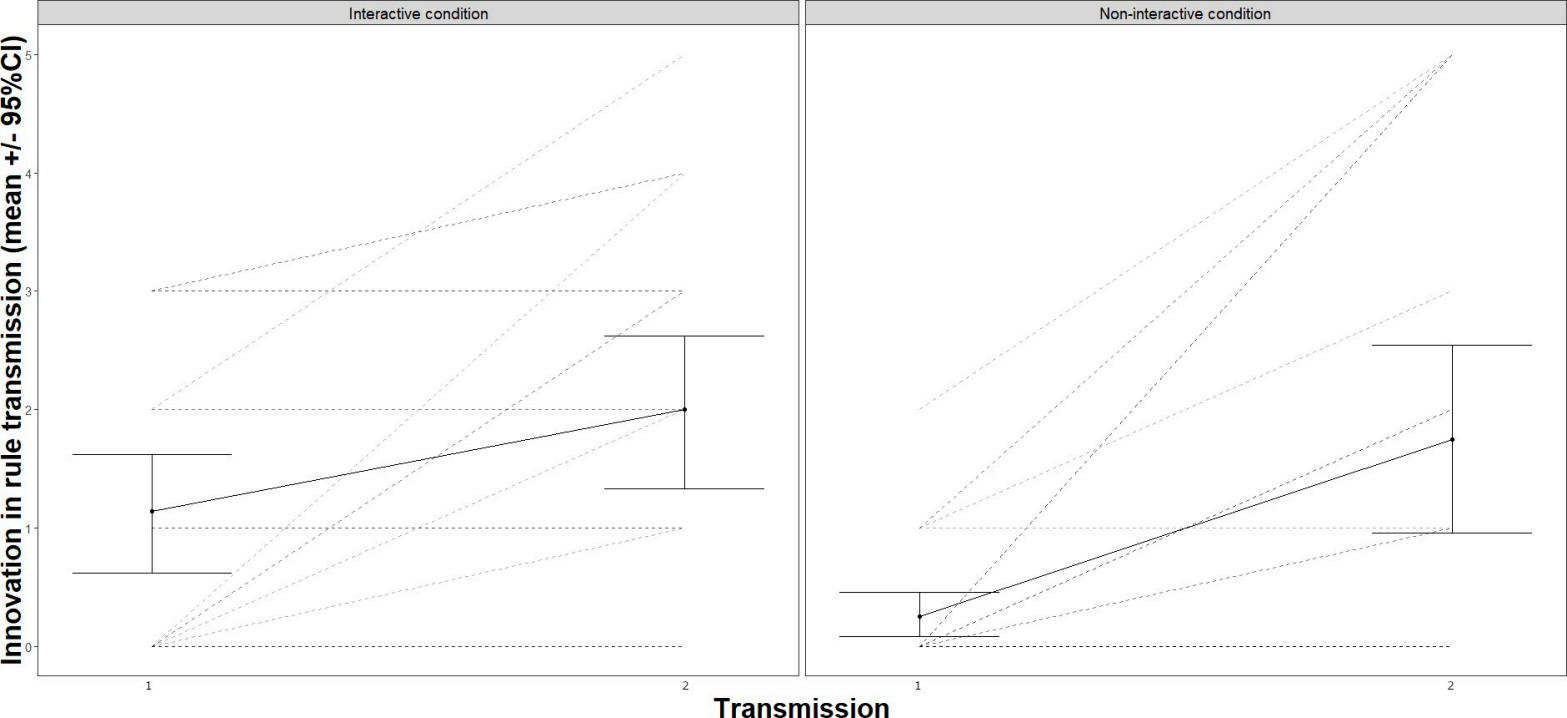
Transmission of innovation about how to use the pasta maker. Zero stands for zero innovation–perfect fidelity to the rule introduced in the video tutorial. Error bars represent standard error to the mean.

In order to test the effect of generation, condition, and previous performance on innovation (H5: The transmission of innovation increased over generations; and H6: Previous performance of teachers predicted the inclusion of innovation in their transmissions) we used a model that included the categorical variables as well as one additional continuous variable (previous performance) as fixed effect and innovation as outcome (Analysis 4, S1B Table). For H5 we observed a significant effect of generation, *F*(1, 40) = 29.16, *p* < .001. That is, innovation reflected in the teachers’ mention of the possibility of using fewer levels of the pasta maker to flatten dough increased over generations (Fig 7). For H6, we observed a significant effect of previous performance, *F*(1, 30) = 7.78, *p* = .009 on the transmission of innovation. In other words, the transmission of innovation increased as previous performance increased, *b* = - 0.42, *SE* = 0.15. In this context increased innovation refers to fact of using less levels of the pasta maker.

We tested the effect of generation, condition, and innovation on follow innovation (H7: Learners followed the innovation transmitted by teachers) with a model that included the categorical variables as well as one additional continuous variable (innovation) as fixed effects and follow innovation as the outcome (Analysis 5, S1B Table). We found that as teachers transmitted innovations to learners, learners implemented those innovations in their performances (follow innovation), *F*(1, 79) = 183.77, *p* < .001. That is, the number of times learners used the pasta maker to flatten the dough decreased as the transmission of innovation increased, *b* = 1.28, *SE* = 0.09. However, we did not observe an effect of innovation on next performance, *F*(1, 26) = 0.02, *p* = .888 (H8: Innovation in teaching sessions predicted increased performance in learners). That is, although learners followed the innovations transmitted by teachers, this did not increase their performance. The same model used to test H4 was employed for testing H8, see above (Analysis 3, S1B Table). The confirmation of H5 and H6 shows that innovations increased over generations, that higher performing learners included more innovations as teachers in their transmission sessions, and that learners followed those innovations.

**Fig 8:**
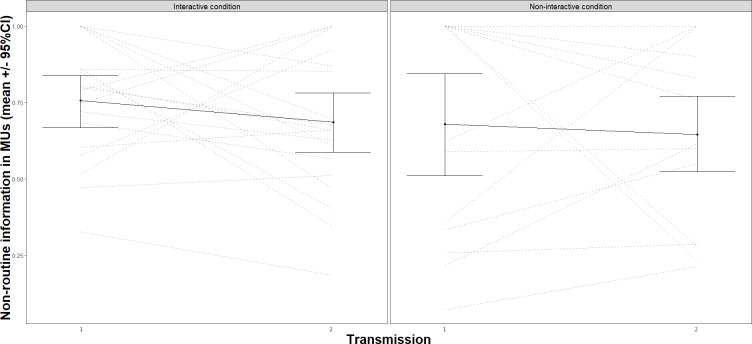
Use of MUs in the transmission of non-routine, unexpected information about the collaborative task. Error bars represent standard error to the mean.

In order to test the effect of generation, condition and MUs on the presence of nonroutine information in transmission sessions (H9: Teachers preferentially use storytelling to transmit non-routine or unexpected information) we used a model that included the same categorical variables as all previous models, as well as one additional categorical variable (in MU) as fixed effects and non-routine information as the outcome (Analysis 6, S1B Table). Storytelling was defined as the transmissions MUs in teaching sessions. For H9, we observed a significant effect of MUs in the transmission of non-routine or unexpected information, *F*(1, 77) = 212.74, *p* < .001 (Fig 8). The model used to test H10 (Teachers’ storytelling increases over generations) and H11 (Teachers’ storytelling is facilitated by interactivity)–effect of generation and condition on MU–included the categorical variables as fixed effects and MU as the outcome (Analysis 7, S1B Table). A significant effect of generation, *F*(1, 71) = 8.99, *p* = .004 confirmed H10 but results did not support H11. That is, MUs increased over generations but interactivity did not increase their inclusion in transmission sessions, *F*(1, 71) = 3.41, *p* = 0.07. These findings show that storytelling was specialized for the transmission of non-routine or unexpected information and that the presence of storytelling increased over generations, but interactivity was not a facilitating factor.

## Discussion

We investigated whether cultural transmission and interactivity affected task performance and its outcomes in a collaborative food preparation task. In our study, we did not detect an effect of generation or interactivity on performance over transmission chains. The fact that we did not find an effect of generation on performance may have been associated with the limited number of generations. The lack of increased performance in the interactive condition may have been related to the particularities of the task: Some materials that the participants had the possibility to use (e.g. ravioli mold; pasta maker and rolling pin) may have operated as cultural affordances [45] already encapsulating relevant information for the successful completion of the task.

We did observe that teachers transmitted more information to learners due to interactivity. That is, they transmitted more information about the task to co-present learners compared to a video camera. This occurred even when they knew that video-recorded instructions would be watched by incoming learners in the transmission chain. This is in line with the idea that interactive dialogue requires pairs to check that they have mutually understood each other (e.g., through the production of feedback and/or repair turns [15, 16, 17]) and that this takes time and additional speech turns. This finding also suggests that teachers did not attempt to “compensate” for the lack of feedback from learners in the non-interactive condition by talking more in the video recordings. Furthermore, these results confirm previous studies using similar methodologies that have investigated the effects of interactivity on the transmission of narrative information only- i.e., participants were only instructed to transmit information not to also perform a manual task [12]. Tan and Fay reported that the transmission of narrative information from narrators (teachers) to listeners (learners) was more accurate because of interactivity, when they interacted freely with one another in transmission chains. This occurred due to the effect of listeners’ behavior, including backchannels or clarification questions. Our study not only confirms Tan and Fay’ findings but it relates them to a more complex task in which participants, apart from transmitting learned information, had to perform a collaborative manual activity.

Our findings showed a clear correspondence between the quantity of information transmitted over generations and increased performance. Increased quantity of information transmission from teachers led to increased performance in learners. These results go beyond the findings reported in previous studies on the positive role of teaching in the transmission of complex manual skills (e.g. tying complicated knots and building stone tools) [23, 46]. They have shown that the same kind of teaching behavior (including verbal and gestural resources) conveyed in different communicative contexts (face-to-face vs. video mediated) has distinct effects on the quantity of information transmitted by teachers and subsequent task performance in learners over transmission chains. This has never been studied in the laboratory before and we believe has important implications for the design of teaching and learning environments.

We further investigated particular features of the information transmitted from teachers to learners. First, we analyzed whether generation and interactivity affected the transmission and implementation of innovations and whether these influenced performance in learners. We observed that (i) the transmission of innovations increased over generations, (ii) better performing generations introduced more innovations as teachers in transmission sessions and that (iii) these were followed by learners when completing the collaborative food preparation task. Our results showed an accumulation of innovations over generations in both transmission and performance sessions. The transmission and accumulation of innovations in transmission chain experiments have rarely been studied before (for a recent study on innovation with young children using an open diffusion design instead of transmission chains see [47]). Previous studies using transmissions chains have generally examined whether generations could successfully copy a given model (in high complexity tasks, e.g., making stone tools [6]) or build an increasingly better performing one (in low complexity tasks; building paper airplanes and measure flight distance, [3]). We observed a correspondence between high performance during the collaborative task and the inclusion of more innovations in subsequent transmission sessions. This finding shows that higher performance generations included more innovation as teachers in transmission sessions. Our results about learners following transmitted innovations was related to two main factors: i) teachers’ transmission of valid and sound justifications (e.g., “in order to save time”) and ii) teachers’ reference to their final performance in terms of number of good quality ravioli.

Finally, we examined whether teachers used storytelling to transmit information about non-routine, uncertain or novel situations to learners (compared to other forms of linguistic communication, e.g., instructional discourse). Storytelling plays a central role in our everyday lives. It is one of the most widespread social activities through which people in different cultures share cultural information [29, 48, 49, 50, 51]. In this study we showed that teachers chose storytelling to transmit non-routine or unexpected information to learners and that this behavior increased over generations. The function of storytelling as a vehicle for the cultural transmission of skills has not been investigated before. Our study showed that storytelling in transmission sessions was a functional tool for making sense of non-routine, novel or uncertain situations. The fact that the specialized role storytelling increased over transmission sessions is in line with previous studies on cumulative cultural evolution in the laboratory showing increased adaptation of functional behaviors over generations [6]. Future experimental studies on cultural transmission, social learning, and cumulative cultural evolution should propose replicable paradigms in terms of task designs and measurements that account for both, low and high complexity manual tasks, at individual and group levels [52]. This would make comparisons across studies and possible meta-analyses more reliable.

In this paper we provided first experimental evidence about i) the effects of interactivity and generation in the transmission of information and how information quantity affected task performance, ii) the emergence and spread of innovations in transmissions chains, and iii) the functional value of storytelling as teaching method. We did it using an everyday activity that is taught and learned across cultures and transmitted over generations in families and group. Future studies on cultural transmission should investigate to what extent the results reported here could be replicated in other collaborative and culturally meaningful tasks.

## Supporting information

S1 Table This document contains a description of the statistical analyses. (DOCX)

## References

[1] O'Brien MJ, Shennan SJ, editors. Innovation in cultural systems Cambridge, MA: MIT Press; 2009.

[2] Mesoudi A, O’Brien M.J. The learning and transmission of hierarchical cultural recipes. Biol. Theory. 2008; 3: 63–72.

[3] Caldwell CA, Millen AE Social learning mechanisms and cumulative cultural evolution: Is imitation necessary? Psychol. Sci. 2009; 20: 1478–1483. 10.1111/j.1467-9280.2009.02469.x 19891752

[4] Kendal RL, Boogert NJ, Rendell L, Laland KN, Webster M, Jones PL. Social learning strategies: Bridge-building between fields. Trends Cogn. Sci. 2018; 22 (7): 651–665. 10.1016/j.tics.2018.04.003 29759889

[5] Kline MA. How to learn about teaching: An evolutionary framework for the study of teaching behavior in humans and other animals. Behav. Brain Sci. 2015; 8 e31 10.1017/S0140525X14000090 24856634

[6] Morgan TJH, Uomini NT, Rendell LE, Chouinard-Thuly L, Street SE, Lewis HM et al Experimental evidence for the co-evolution of hominin tool-making teaching and language. Nat. Commun. 2015; 6 6029 10.1038/ncomms7029 25585382 PMC4338549

[7] Lombao D, Guardiola M, Mosquera M. Teaching to make stone tools: new experimental evidence supporting a technological hypothesis for the origins of language. Sci. Rep. 2017; 14394 10.1038/s41598-017-14322-y PMC5663762 29089534

[8] Hochberg ME, Marquet PA, Boyd R, Wagner A. Innovation: an emerging focus from cells to societies. Philos. Trans. R. Soc. B, Bil Sci. 2017; 372(1735): pii: 20160414. 10.1098/rstb.2016.0414 29061887 PMC5665802

[9] Bavelas JB, Coates L, Johnson T. Listeners as co-narrators. J. Pers, Soc. Psychol. 2000; 79: 941–942. 11138763 10.1037//0022-3514.79.6.941

[10] Dunbar RIM. Gossip in evolutionary perspective. Rev. Gen. Psychol. 2004; 8: 100–110.

[11] Maswood R, Rajaram S. Social transmission of false memory in small groups and large networks. Top. Cogn. Sci. 2018 10.1111/tops.12348 29785724

[12] Tan R, Fay N. Cultural transmission in the laboratory: Agent interaction improves the intergenerational transfer of information. Evol. Hum. Behav. 2011; 32: 399–406.

[13] Fay N, Ellison TM, Tylén K. Fusaroli R, Walker B, Garrod S. Cultural ratchet to a social artefact: The cumulative cultural evolution of a language game. Evol. Hum. Behav. 2018; 39: 300–309.

[14] Clark HH. Using language Cambridge, MA: Cambridge University Press; 1996.

[15] Clark HH, Wilkes-Gibbs D. Referring as a collaborative process. Cognition. 1986: 22, 1‑39. 10.1016/0010-0277(86)90010-7. 3709088

[16] Clark HH, Schaefer EF. Contributing to discourse. Cogn. Sci. 1989; 13: 259‑294. 10.1207/s15516709cog1302_7.

[17] Clark HH, Brennan SE. Grounding in communication In: Resnick LB, Levine JM, Teasley SD, editors. Perspectives on socially shared cognition. Washington, DC: American Psychological Association; 1991, pp.127–149.

[18] Knutsen D, Ros C, Le Bigot L. Generating references in naturalistic face-to-face and phone mediated dialogue settings. Top. Cogn. Sci. 2016; 8: 796‑818. 10.1111/tops.12218 27541074

[19] Kraut RE, Fussell SR, Siegel J. Visual information as a conversational resource in collaborative physical tasks. Hum. Comput. Interact. 2003; 18: 13–49. 10.1207/S15327051HCI1812_2

[20] Caldwell CA, Atkinson M, Renner E. Experimental approaches to studying cumulative cultural evolution. Curr. Dir. Psychol, Sci, 2016; 25: 191–195. 27397972 10.1177/0963721416641049 PMC4936513

[21] Mesoudi A, Whiten A. The multiple roles of cultural transmission experiments in understanding human cultural evolution. Philos. Trans. R. Soc. B, Bil Sci. 2008; 363, 3489–3501. 10.1098/rstb.2008.0129 PMC2607337 18801720

[22] Bartlett F. Remembering. Cambridge: Cambridge University Press; 1932.

[23] Zwirner E, Thornton A. Cognitive requirements of cumulative culture: teaching is useful but not essential. Sci. Rep. 2015; 5: 16781 10.1038/srep16781 26606853 PMC4660383

[24] Caldwell CA, Millen A. Experimental models for testing hypotheses about cumulative cultural evolution. Evol. Hum. Behav. 2008; 29: 165–171.

[25] Bietti LM, Tilston OER, Bangerter A. Storytelling as adaptive collective sensemaking. Top. Cogn. Sci. 2018; 10.1111/tops.12358. PMC7379714 29954043

[26] Mandelbaum J. (2013). Storytelling in conversation In: Sidnell J, Stivers T, editors. Handbook of conversation analysis. Cambridge, UK: Cambridge University Press; 2013, pp. 492–508.

[27] Labov W, Waletzky J. Narrative analysis In: Helm J. editor. Essays on the verbal and visual arts. Seattle: University of Washington Press; 1976, pp. 12–44.

[28] Bruner JS. Acts of meaning Cambridge, MA: Harvard University Press; 1990

[29] Scalise Sugiyama M. Food, foragers, and folklore: The role of narrative in human subsistence. Evol. Hum. Behav. 2001; 22: 221–240

[30] Brown DE. Human Universals. New York: McGraw-Hill; 1991.

[31] Smith D, Schlaepfer P, Major K, Dyble M, Page AE., Thompson J, et al. Cooperation and the evolution of hunter-gatherer storytelling. Nat. Comm. 2017; 8: 1–19. 1853. 10.1038/s41467-017-02036-8. PMC5717173 29208949

[32] Garfield ZH, Garfield MJ, Hewlett BS. A cross-cultural analysis of hunter-gatherer social learning In: Terashima H, Hewlett B, editors. Social learning and innovation in contemporary hunter-gatherers. Tokyo, Japan: Springer; 2016, pp. 19–34. 10.1007/978-4-431-55997-9_2

[33] Silva KG, Correa-Chávez M, Rogoff B. Mexican-heritage children's attention and learning from interactions directed to others. Child Dev. 2010; 81: 898–912. 10.1111/j.1467-8624.2010.01441.x 20573112

[34] Dessalles JL. (2010). Have you anything unexpected to say? The human propensity to communicate surprise and its role in the emergence of language In: Smith ADM, Schouwstra M, de Boer B, Smith K. editors. The evolution of language. Proceedings of the 8th international conference (Evolang8—Utrecht). Singapore: World Scientific; 2010, pp.99–106. 10.1142/9789814295222_0013

[35] Bietti LM, Bangerter A, Mayor E. The interactive shaping of social learning in transmission chains In: Gunzelmann G, Howes A, Tenbrink T, Davelaar E, editors. Proc. 39th Annual Conf. Cognitive Science Soc. Austin, TX: Cognitive Science Society; 2017, pp.1641–1646.

[36] Aiello LC, Wheeler P. The expensive-tissue hypothesis—The brain and the digestive system in human and primate evolution. Curr Anthropol. 1995;36:199–221. 10.1086/204350

[37] Carmody RN, Wrangham RW. The energetic significance of cooking. J Hum Evol. 2009; 57:379–391. 10.1016/j.jhevol.2009.02.011 19732938

[38] McCabe M, de Waal Malefyt T. Creativity and cooking: Motherhood, agency and social change in everyday life. J Consum Cult. 2015; 15: 48–65.

[39] Farmer N, Touchton-Leonard K, Ross A. Psychosocial benefits of cooking interventions: a systematic review. Health Educ Behav. 45: 167–80. 10.1177/1090198117736352 29121776 PMC5862744

[40] Wrangham RW, Jones JH, Laden G, Pilbeam D, Conklin-Brittain N. The raw and the stolen—Cooking and the ecology of human origins. Curr Anthropol. 1999;40:567–594. 10.1086/300083 10539941

[41] Utter J, Larson N, Laska MN, Winkler M, Neumark-Sztainer D. Self perceived cooking skills in emerging adulthood predict better dietary behaviors and intake 10 years later: a longitudinal study. J Nutr Educ Behav. 2018; 50: 494–500. 10.1016/j.jneb.2018.01.021 29525525 PMC6086120

[42] Boyd R, Richerson PJ. Culture and the evolutionary process Chicago: University of Chicago Press; 1985.

[43] Derex M, Boyd R, The foundations of the human cultural niche. Nat. Commun. 2015; 6, 8398 10.1038/ncomms9398 26400015 PMC4598620

[44] Bangerter A. Identifying individual and collective acts of remembering in task-related communication. Discourse Proces. 2000; 30: 237–264.

[45] Ramstead MJD, Veissière SPL, Kirmayer LJ. Cultural affordances: Scaffolding local worlds through shared intentionality and regimes of attention. Front Psychol. 2016; 7:1090 10.3389/fpsyg.2016.01090 27507953 PMC4960915

[46] Caldwell CA., Renner E, Atkinson M. Human teaching and cumulative cultural evolution. Rev. Phil. Psych. 2018; 9: 751 10.1007/s13164-017-0346-3 30595765 PMC6290649

[47] McGuigan N, Burdett E., Burgess V, Dean L, Lucas A, Vale G et al Innovation and social transmission in experimental micro-societies: exploring the scope of cumulative culture in young children. Philos. Trans. R. Soc. B, Bil Sci. 2017; 5;372(1735). pii: 20160425 10.1098/rstb.2016.0425 29061897 PMC5665812

[48] Bangerter A, Mayor E, Pekarek Doehler S. Reported speech in conversational storytelling during nursing shift handover meetings. Discourse Proces. 2011; 48: 183–214. 10.1080/0163853X.2010.519765.

[49] Boyd B. On the origins of stories: Evolution, cognition and fiction Cambridge, MA: Harvard University Press; 2009.

[50] Currie A, Sterelny K. (2017). In defence of story‐telling. Stud. His. Philos. Sci. A. 2017; 62: 14–21. 10.1016/j.shpsa.2017.03.003. 28583355

[51] Dunbar R. Grooming, gossip and the evolution of language London: Faber and Faber; 2010.

[52] Milton H, Charbonneau M. Cumulative culture in the laboratory: methodological and theoretical challenges. Philos. Trans. R. Soc. B, Bil Sci. 2018; 285(1879): 20180677 10.1098/rspb.2018.0677 PMC5998114 29848653

