# Supplementary material for: Cultural transmission in a food preparation task: The role of interactivity, innovation and storytelling
Source: PLoS One. 2019 Sep 18;14(9):e0221278. doi: 10.1371/journal.pone.0221278 (PMC6750589; doi:10.1371/journal.pone.0221278)
Supplement: S1 Table — (DOCX) [file pone.0221278.s001.docx]

**Supporting information**

**Cultural transmission in a food preparation task: The role of interactivity, innovation and storytelling**

Lucas M. Bietti^1, 2 *^, Adrian Bangerter ^1^, Dominique Knutsen ^3^, Eric Mayor ^1^

^1^ Institute of Work and Organizational Psychology, University of Neuchâtel, Neuchâtel, Switzerland

^2^ Centre National de la Recherche Scientifique (CNRS), Télécom Paris, Institut Interdisciplinaire de l’innovation, UMR 9217, Paris, France

^3^ Univ. Lille, CNRS, CHU Lille, UMR 9193 - SCALab - Sciences Cognitives et Sciences

Affectives, F-59000 Lille, France

* Corresponding author

E-mail: [bietti@protonmail.com](mailto:bietti@protonmail.com) (LMB)

**Statistical analysis**

The data were analyzed using linear mixed models, accounting for the inclusion of generations within groups. To begin with, we attempted to implement the maximal random effects structure justified by the design in each model [1]. Whenever this caused convergence issues, the random effects causing these issues were identified (this is done automatically in SAS [2, 3]), removed, and the analysis was conducted again. The final random effects structure used in the analysis is reported below. The degrees of freedom of the models were corrected using the Satterthwaite approximation, as the number of observations was not always the same in each cell of the design in particular due to missing observations.

Table A. Random Effects Implemented in the Analyses

| **Analysis** | **Random effects structure implemented** |
| --- | --- |
| 1 | By-group random intercepts, by-group random slopes corresponding to generation |
| 2 | By-group random intercepts, by-group random slopes corresponding to generation |
| 3 | By-group random intercepts, by-group random slopes corresponding to generation |
| 4 | By-group random intercepts, by-group random slopes corresponding to generation and previous performance |
| 5 | By-group random intercepts, by-group random slopes corresponding to generation |
| 6 | By-group random slopes corresponding to non-routine or unexpected events in MUs |
| 7 | By-group random intercepts, by-group random slopes corresponding to generation |

Table B. Results of Models 1 to 7^[[1]](#footnote-1)^.

| **Analysis** | **Outcome variable** | **Fixed effect** | ***b*** | ***SE*** | ***p*** |
| --- | --- | --- | --- | --- | --- |
| 1 | Previous performance | Generation: 2 | -2.54 | 2.69 | .348 |
|  |  | Generation: 3 | -0.67 | 2.69 | .805 |
|  |  | Generation: 1 | 0 |  |  |
|  |  | Condition: Interactive | -4.06 | 3.00 | .178 |
|  |  | Condition: Non-interactive | 0 |  |  |
|  |  | Condition: Interactive x Generation: 2 | 4.35 | 3.94 | .273 |
|  |  | Condition: Interactive x Generation: 3 | 7.00 | 3.94 | .079 |
|  |  | Condition: Interactive x Generation: 1 | 0 |  |  |
|  |  | Condition: Non-interactive x Generation: 2 | 0 |  |  |
|  |  | Condition: Non-interactive x Generation: 3 | 0 |  |  |
|  |  | Condition: Non-interactive x Generation: 1 | 0 |  |  |
| 2 | Information quantity | Generation: 2 | -64.00 | 59.97 | .292 |
|  |  | Generation: 1 | 0 |  |  |
|  |  | Condition: Interactive | 316.04 | 84.53 | < .001 |
|  |  | Condition: Non-interactive | 0 |  |  |
|  |  | Condition: Interactive x Generation: 2 | 125.52 | 87.79 | .160 |
|  |  | Condition: Interactive x Generation: 1 | 0 |  |  |
|  |  | Condition: Non-interactive x Generation: 2 | 0 |  |  |
|  |  | Condition: Non-interactive x Generation: 1 | 0 |  |  |
| 3 | Next performance | Condition: Interactive | 1.11 | 3.49 | .752 |
|  |  | Condition: Non-interactive | 0 |  |  |
|  |  | Generation: 3 | -4.45 | 3.02 | .146 |
|  |  | Generation: 2 | 0 |  |  |
|  |  | Condition: Interactive x Generation: 3 | 0.30 | 4.21 | .944 |
|  |  | Condition: Interactive x Generation: 2 | 0 |  |  |
|  |  | Condition: Non-interactive x Generation: 3 | 0 |  |  |
|  |  | Condition: Non-interactive x Generation: 2 | 0 |  |  |
|  |  | Information quantity | 3.01 | 1.32 | .026 |
|  |  | Innovation | -0.20 | 1.40 | .888 |
|  |  | MUs in innovation | -0.75 | 1.14 | .521 |
|  |  | Advice in innovation | -1.60 | 1.15 | .170 |
| 4 | Innovation | Condition: Interactive | 0.24 | 0.44 | .593 |
|  |  | Condition: Non-interactive | 0 |  |  |
|  |  | Generation: 2 | 0.78 | 0.32 | .021 |
|  |  | Generation: 1 | 0 |  |  |
|  |  | Condition: Interactive x Generation: 2 | 0.83 | 0.45 | .071 |
|  |  | Condition: Interactive x Generation: 1 | 0 |  |  |
|  |  | Condition: Non-interactive x Generation: 2 | 0 |  |  |
|  |  | Condition: Non-interactive x Generation: 1 | 0 |  |  |
|  |  | Previous performance | -0.42 | 0.15 | .009 |
| 5 | Follow innovation | Condition: Interactive | -0.31 | 0.24 | .193 |
|  |  | Condition: Non-interactive | 0 |  |  |
|  |  | Generation: 3 | -0.11 | 0.23 | .640 |
|  |  | Generation: 2 | 0 |  |  |
|  |  | Condition: Interactive x Generation: 3 | 0.33 | 0.32 | .295 |
|  |  | Condition: Interactive x Generation: 2 | 0 |  |  |
|  |  | Condition: Non-interactive x Generation: 3 | 0 |  |  |
|  |  | Condition: Non-interactive x Generation: 2 | 0 |  |  |
|  |  | SumTransST | 1.28 | 0.09 | < .001 |
| 6 | Non-routine information | Condition: Interactive | 0.06 | 0.04 | .178 |
|  |  | Condition: Non-interactive | 0 |  |  |
|  |  | Generation: 2 | 0.01 | 0.04 | .877 |
|  |  | Generation: 1 | 0 |  |  |
|  |  | Condition: Interactive x Generation: 2 | -0.02 | 0.06 | .719 |
|  |  | Condition: Interactive x Generation: 1 | 0 |  |  |
|  |  | Condition: Non-interactive x Generation: 2 | 0 |  |  |
|  |  | Condition: Non-Interactive x Generation: 1 | 0 |  |  |
|  |  | In MU: 1 | 0.45 | 0.03 | < .001 |
|  |  | In Non-MU: 0 | 0 |  |  |
| 7 | Memory utterance (MU) | Condition: Interactive | 0.05 | 0.03 | .048 |
|  |  | Condition: Non-interactive | 0 |  |  |
|  |  | Generation: 2 | 0.08 | 0.03 | .006 |
|  |  | Generation: 1 | 0 |  |  |
|  |  | Condition: Interactive x Generation: 2 | -0.04 | 0.04 | .295 |
|  |  | Condition: Interactive x Generation: 1 | 0 |  |  |
|  |  | Condition: Non-interactive x Generation: 2 | 0 |  |  |
|  |  | Condition: Non-Interactive x Generation: 1 | 0 |  |  |

Each analysis always included generation, condition, and the interaction between these two factors, either because this was required by the hypothesis, or in order to control for these categorical variables. Each analysis could also include one or several numerical variables, depending on the hypothesis tested. Importantly, in some cases, two hypotheses or more involved the same dependent variable (for instance, both H5 and H6 involved the DV Innovation). In such situations, this DV was analyzed using a single model which included any other independent variable (IV) named in the hypotheses (e.g., the single model used to analyze the DV Innovation included the IVs generation [in order to test H5] and the IV Previous performance [in order to test H6]^[[2]](#footnote-2)^). Doing so enabled us to examine the effect of each IV on the DV while all the while controlling for the effect of all other IVs of interest.

All numeric variables were standardized to enable us to interpret the *b* coefficients correctly. For the same reason, categorical variables were coded as orthogonal contrasts whenever a model included both categorical and continuous variables. Interactions between the categorical variables and the continuous variables were not included because no hypothesis was formulated regarding them. Graphs were conducted with the data visualization package ggplot2 in R [4].

**References**

1. Barr DJ, Levy R, Scheepers C, Tilly, HJ. Random effects structure for confirmatory hypothesis testing: Keep it maximal. *J Mem Lang*. 2013; 68: 255–278.

2. Kiernan K, Tao J, Gibbs P. Tips and strategies for mixed modeling with SAS/STAT procedures. SAS Global Forum, Orlando, FL; 2012. Available from: https://support.sas.com/resources/papers/proceedings12/332-2012.pdf

3. Knutsen D, Le Bigot L. The influence of reference acceptance and reuse on conversational memory traces. *J Exp Psychol Learn Mem Cogn*. 2015; 41: 574-585.

4. Wickham H, Chang W. ggplot2: An implementation of the Grammar of Graphics. *R package version 0.7* (2008) URL: <http://CRAN.R-project>. org/package= ggplot2.

1. As mentioned in the main manuscript, the F value associated with the main effect of condition in analysis 7 is non-significant. The parameters reported in this table seems to contradict this, as the p value associated with the b coefficient for condition is .048. This is because in this kind of analysis, the significance level of b coefficients reflect the significance level of the effect present in the model, whereas F values reflect the effect overall. In other words, because there is an interaction in this model, the b coefficient associated with condition is significant in the reference category of the interaction. However, it seems that this effect was not strong enough (a) to make the overall F-value associated with condition significant or (b) to make the interaction significant. [↑](#footnote-ref-1)
2. It also included the IV condition, as well as the interaction between condition and generation, because, as mentioned above, these were included in all models. [↑](#footnote-ref-2)
